# Attachment Representations and Brain Asymmetry during the Processing of Autobiographical Emotional Memories in Late Adolescence

**DOI:** 10.3389/fnhum.2016.00644

**Published:** 2016-12-26

**Authors:** Melanie T. Kungl, Rainer Leyh, Gottfried Spangler

**Affiliations:** Institute of Psychology, Friedrich-Alexander-Universität Erlangen-NürnbergErlangen, Germany

**Keywords:** attachment, EEG, hemispheric asymmetry, emotional memory, early adulthood

## Abstract

Frontal and parietal asymmetries have repeatedly been shown to be related to specific functional mechanisms involved in emotion regulation. From a developmental perspective, attachment representations based on experiences with the caregiver are theorized to serve regulatory functions and influence how individuals deal with emotionally challenging situations throughout the life span. This study aimed to investigate neural substrates of emotion regulation by assessing state- and trait dependent EEG asymmetries in secure, insecure-dismissing and insecure-preoccupied subjects. The sample consisted of 40 late adolescents. The Adult Attachment Interview was administered and they were asked to report upon personally highly salient emotional memories related to anger, happiness and sadness. EEG was recorded at rest and during the retrieval of each of these emotional memories, and frontal and parietal hemispheric asymmetry were analyzed. We found attachment representations to differentially affect both the frontal and parietal organization of hemispheric asymmetry at rest and (for parietal region only) during the retrieval of emotional memories. During rest, insecure-dismissing subjects showed an elevated right-frontal brain activity and a reduced right-parietal brain activity. We interpret this finding in light of a disposition to use withdrawal strategies and low trait arousal in insecure-dismissing subjects. Emotional memory retrieval did not affect frontal asymmetry. However, both insecure groups showed an increase in right-sided parietal activity indicating increased arousal during the emotional task as compared to the resting state suggesting that their emotion regulation capability was especially challenged by the retrieval of emotional memories while securely attached subjects maintained a state of moderate arousal. The specific neurophysiological pattern of insecure-dismissing subjects is discussed with regard to a vulnerability to affective disorders.

## Introduction

A central tenet of attachment theory is that depending on the caregiver’s availability and responsiveness children form expectations about how they can rely on the caregiver in the face of stress and consequently develop individual ways to regulate behavior in emotionally challenging situations (Bowlby, 1969; Ainsworth et al., 1974). For example, when the caregiver poorly or inappropriately responds to the child’s signals of distress, the child may learn to avoid expressing negative affect in the future. These experiences are believed to result in a so-called inner working model of attachment that serves regulatory functions throughout the life span (Bowlby, 1982; Bretherton and Munholland, 2008). Applying a developmental perspective, the current study set out to investigate how in late adolescence working models of attachment influence hemispheric brain activity related to emotional processing at rest and during the retrieval of autobiographic emotional events that have occurred beyond childhood. This research is important, as biological substrates of emotion regulation capacities may reveal dysfunctional processes that are not inferable via narratives or self-report. Importantly, in adolescence, the generation of a meaningful life story emerges Habermas and Bluck, 2000. The effective conscious recollection of personally salient emotional memories plays a major part in this development and is linked to psychological growth and well-being (Singer et al., 2013). Moreover, the “the updating of prior emotional memories through a process of reconsolidation” can be seen as a core element of different psychotherapeutic approaches as it enables the incorporation of new experiences, and thus, supports healthy development (Lane et al., 2015, p. 1). With this study we aim to add to current knowledge on processes involved in emotional memory retrieval with special regard to individual differences related to attachment.

While in early childhood individual patterns of attachment come to light by assessing children’s behavioral organization in stressful situations (e.g., the Strange Situation Procedure, Ainsworth and Wittig, 1969) with increasing age, behavioral differences become more subtle. By late childhood, inner working models of attachment are believed to be reflected in narratives about attachment related topics, and would typically be assessed by using interview measures. The Adult Attachment Interview (AAI, Main et al., 2002) reveals classifications of attachment that are based on explicit assumptions about securely, insecure-dismissing and insecure-preoccupied individuals’ way to access and regulate their emotions. As theorized by Main et al. (1985) when asked about his or her experiences with the caregiver, a securely attached person is capable of freely evaluating and openly communicating pleasant and unpleasant emotions. Irrespective of their valence, he or she can reflect upon his or her experiences and integrate them as important aspects of the past and current self. In contrast, insecurely attached persons are unable to present a coherent life history with regard to attachment related issues. Presumably due to having experienced an irresponsive caregiver that did not provide the opportunity to communicate various aspects of emotions, insecure-dismissing individuals appear to only have limited access to their feelings and are less capable of perceiving and expressing emotional information. As a consequence, using a rather unconscious defensive process, they tend to idealize attachment experiences or present them as less meaningful to their development. In contrast to these de-activating strategies, insecure-preoccupied individuals seem to be overwhelmed by their emotions, especially regarding anger. At the same time they make contradictory statements leading to an incoherent narrative reflective of their deficits to integrate conflicting emotions. Instead they appear to be ambivalent, entangled and highly affected when reporting about their past. The insecure-preoccupied state of mind is thought to be a result of inconsistent responsiveness of the caregiver during the early years (Cassidy and Berlin, 1994).

### Attachment and Emotion Regulation

Furthermore, it is assumed that regulatory capacities of grown-up individuals with different attachment representations not only apply to the retrieval of childhood experiences but that they also result in a certain predisposition to respond to various kinds of emotional challenges (Spangler and Zimmermann, 1999; Cassidy, 2008). As such, inner working models of attachment affect, for example, how emotional stimuli are perceived and interpreted and, by doing so, shape how the individual experiences his or her inner and outer world, hence guiding subsequent behavior. Indeed, insecurely attached individuals have been found to show deficits in the processing of emotional stimuli. For example, studies using self-report of attachment have found heightened or reduced attention in insecure-ambivalent^1^ and insecure-avoidant subjects, respectively, when compared to secure ones (Hazan and Shaver, 1994; Simpson et al., 1996; Cooper et al., 1998). Using the AAI, Spangler and Zimmermann (1999) found insecure-dismissing adolescents failing to show typical mimic responses to emotional film clips, indicating reduced emotional expressiveness. Interestingly, on a declarative level they were also found to report reduced attention to negative stimuli (Spangler and Zimmermann, 1999). Also, empirical evidence suggests that insecurely attached mothers are less accurate in identifying infant emotion and process infants’ negative emotional expressions in a specific way (e.g., Spangler et al., 2010). In this line, a number of ERP studies report evidence of attachment related differences in the neural processing of emotional stimuli. For example, analyzing ERP responses to infant emotional faces studies using narrative measures of attachment found neural correlates associated with attentional processing (i.e., N200, P3) to be less prominent in insecure mothers (Fraedrich et al., 2010; Leyh et al., 2016a). Accordingly, studies using self-report measures of attachment found decreased ERP amplitudes in response to emotional faces in avoidant subjects (Zhang et al., 2008), as well as a response bias in favor of positive stimuli (Chavis and Kisley, 2012), while others found insecurely attached individuals to be less able to accurately discriminate between different facial emotion expressions on a neurophysiological level (Escobar et al., 2013; for a review also see Gander and Buchheim, 2015).

There also is evidence for attachment differences in adolescents’ emotion regulation behavior and adrenocortical regulation in specific problem-solving and emotion eliciting tasks (e.g., Zimmermann et al., 2001; Spangler and Zimmermann, 2014) as well as in individuals’ physiological reactivity during family conflict interaction (Beijersbergen et al., 2008). For example, Beijersbergen et al. (2008) found insecure-dismissing subjects to elicit increased heart rate reactivity when their emotion regulation strategies were challenged during the interaction, however, their defensive mechanisms seem to work well during the retrieval of caregiving experiences (but see Roisman et al., 2004). Taken together, including physiological measures when investigating psychological processes appears to reveal insecurely attached individuals’ struggle to effectively regulate affective states which would not be accessible by verbal communication only.

In sum, these findings, together with numerous other studies provide strong evidence that patterns of attachment account for variability in adolescents’ and adults’ emotion regulation strategies on both a behavioral as well as a psycho-biological level (also see Spangler and Zimmermann, 1999; Gander and Buchheim, 2015).

### The Role of Hemispheric Asymmetries in Emotion Regulation

During the last decade the use of EEG as a measure of the brain’s responses to emotional stimuli and its activity during emotional tasks has gained great popularity. To date, there is a still increasing number of neurophysiological studies investigating the neural circuits underlying differences regarding securely and insecurely attached individuals’ processing of emotions. One common approach in the EEG literature is the use of EEG asymmetries reflecting the functional involvement of both hemispheres during an extended recording episode which is associated with well-grounded theoretical models. Beside the measurement of individual affective dispositions, EEG asymmetries are also modulated by, for example, the induction of certain emotions, thus, serving as a trait as well as a state variable, respectively (e.g., Coan and Allen, 2003). Regarding both of these aspects of emotion regulation, ongoing alpha power^2^ (8–13 Hz) at rest and its moderation by emotional content are the major focus of the current study.

Left and right frontal cerebral regions have shown to be differentially involved in the processing of different types of emotion (e.g., Coan and Allen, 2004). More precisely, relatively increased left frontal brain activity (LFA) is conceived as being associated with positive affect and increased approach-oriented behaviors. On the other hand, relatively increased right frontal brain activity (RFA) is thought to reflect a motivational tendency to use withdrawal strategies to regulate emotions and is associated with negative affect (Davidson and Fox, 1982; Davidson, 1993). The model has repeatedly been confirmed by studies in both infants and adults (see Davidson, 2004; Marshall and Fox, 2007).

While there has been considerable research on frontal EEG asymmetry, somewhat less is known about the role of parietal brain asymmetry in emotion processing. However, EEG asymmetry scores at parietal cortical areas –with special regard to the right hemisphere- are assumed to reflect additional aspects of the neural processing of emotions, namely the arousal component related to affective states (Heller, 1990). For example, involving a posterior brain system enhanced arousal is thought to typically elicit relatively increased right parietal activity (RPA) (Heller et al., 1997). Furthermore, the role of relatively decreased RPA as a psychophysiological indicator of risk for depression is strongly undermined by empirical evidence (reviewed in Stewart et al., 2011). These findings go along well as they suggest reduced RPA in depressed individuals to indicate low emotional arousal, which itself is commonly associated with the disorder (Mennella et al., 2015). To sum up, both frontal and parietal regions seem to be involved in emotion regulation; however, they appear to be distinctively related to different components of affect.

### Attachment and Hemispheric Asymmetries

To date, there are a few studies that have investigated the association between attachment and EEG asymmetries. However, most of them refer to asymmetrical patterns of ERP waveforms elicited during emotion perception (for a review see Gander and Buchheim, 2015). As an exception, in her infant study, Dawson et al. (2001) found that in interaction with their mothers insecurely attached infants exhibited reduced LFA, which is interpreted in line with a hypo-activation of the attachment system. Investigating this linkage in an adult sample, Rognoni et al. (2008) identified specific patterns of frontal cerebral asymmetry varying as a function of attachment style assessed by questionnaire. In particular, they found attachment insecurity to be associated with greater RFA and security with greater LFA in a resting state indicating avoidance and approach motivation, respectively. Furthermore, attachment groups, on a neural level, differentially responded to emotional stimuli (Rognoni et al., 2008). Accordingly, using the Adult Attachment Projective, Fraedrich et al. (2010) reported further evidence suggesting increased RFA in insecurely attached subjects, however, they could not replicate Rognoni et al.’s (2008) findings with regard to statistical significance (Fraedrich et al., 2010). Regarding attachment and hemispheric asymmetry in parietal regions, Rognoni et al. (2008) briefly reported not to have found any effects. Apart from this, to the best of our knowledge there is no study investigating attachment related differences with regard to parietal asymmetry.

Regarding task specificity, it is suggested, that associations between an individual’s emotion-regulatory capability and hemispheric asymmetries can best be inferred by assessing EEG within an emotional context (e.g., Coan and Allen, 2003; Dennis and Solomon, 2010) or by inducing an affective state as recommended by Mennella et al. (2015). Drawing on a number of inconsistent findings regarding both frontal and parietal asymmetries, they summarize several studies indicating that resting state assessments may not be powerful enough to reliably elicit individual differences in brain activity. Furthermore, they suggest using emotional tasks that especially activate brain regions of interest. In particular, differences in the parietal region may best be observed when using imagery tasks (Mennella et al., 2015). From an attachment theory perspective, the attachment system gets activated in emotionally challenging situations. Thus, it would only be plausible that differences in EEG asymmetries especially come to light during affect regulation. With attachment security as being reflected in a coherent report of one’s attachment history (Main et al., 1985), we assumed that differences in asymmetrical brain activity may be most prominent when inducing an affective state that is linked to highly salient autobiographical memories. While there is evidence, that depending on their mental representation of attachment security, individuals elicit different psychophysiological reaction when talking about their childhood experiences (Roisman et al., 2004), we are not aware of any study that has investigated this effect using EEG measures when processing personally meaningful experiences.

Furthermore, it should be noted that many EEG studies on attachment rely on self-report measures, however, they assess different aspects of attachment than interview measures that take into account mental processes operating on a rather subconscious level. Indeed, correlations between these two measurements is only small (Roisman et al., 2007). Possibly due to the fact that the AAI as well as the EEG are both time consuming methods and, in addition, both require particular expertise for analysis, neurophysiological studies using narratives to assess individual representations of attachment are rather sparse. This void in the literature is addressed in the current study.

### Hypotheses

Drawing from empirical evidence and theoretical assumptions reported above, we expected hemispheric asymmetries to be affected by an individual’s attachment representation at rest as well as during the retrieval of personally salient emotional memories (resembling a trait and a state marker, respectively).

Regarding frontal asymmetry at rest we hypothesized that insecure-dismissing attached subjects would show relatively increased RFA linked to a motivational tendency of withdraw and avoidance (Davidson, 2004) as compared to securely attached ones. With regard to parietal asymmetry linked to arousal (Heller et al., 1997), we hypothesized that insecure-dismissing subjects would show a pattern of hypo-arousal as indicated by reduced RPA while insecure-preoccupied subjects, assumed to have a lower threshold to distress, may show the opposite pattern. For securely attached subjects, however, we expected a more regulated pattern falling in between the hypo- and hyper-activating disposition expected in the insecure-dismissing and the insecure-preoccupied group, respectively.

Furthermore, our study particularly aimed to investigate attachment related differences in state dependent hemispheric asymmetry patterns. In this regard we expected that the retrieval of personally salient emotional memories would affect brain activity patterns, and, as attachment is associated with how individuals regulate their affective states we expected the effect to vary as a function of attachment. Thus, when comparing brain activity at rest (trait) to emotional memory retrieval (state), we hypothesized that insecurely attached subjects would show increased RPA indicating increased arousal during emotional memory retrieval as compared to hemispheric parietal activity at rest. This pattern reflecting a restricted capability to effectively regulate their affective states was not expected in securely attached subjects.

## Materials and Methods

### Participants

The sample consisted of fourty-two late adolescents (22 female, 20 male) ranging from 17 to 22 years of age (*M* = 19.46, *SD* = 1.27). Participants were recruited with flyers to take part in a larger study including three laboratory visits. They were compensated 40 Euros in total. The current paper refers to data collected at the first and second laboratory assessment^3^.

Right-handedness was a selection criterion and all participants completed the Edinburgh Handedness Inventory (EHI, Oldfield, 1971) upon arrival. Twenty-nine percent of the participants were currently attending school while the rest has already graduated but was not yet enrolled at University. Two participants were excluded from further analysis due to non-compliance with the study procedure and insufficient artifact-free EEG data. Thus, data from 40 participants were used for statistical analysis.

### Procedure

On arrival participants gave written consent. The first laboratory visit included the assessment of attachment representations, handedness and autobiographical emotional experiences. Within a few weeks, at the second laboratory visit, neurophysiological data were collected using stimuli extracted from participants’ specifications about their emotional experiences reported earlier. Each laboratory assessment lasted approximately 2.5 h.

### Materials

#### Assessment of Attachment Representations

Attachment representation was assessed by the AAI (George et al., 1985), a semi-structured interview focusing on significant caregiving experiences and attachment relevant situations in childhood. Furthermore, it targets the evaluation of these experiences as well as the current relationship to the primary caregivers^4^. Transcripts of these interviews were coded in accordance with Main et al. (2002). The judgment of narrative coherence, idealization and derogation of parents and/or attachment, as well as current preoccupying anger and passivity of speech results in one of the three main attachment categories: Secure (F), Insecure-Dismissing (Ds), Insecure-Preoccupied (E). The AAI’s reliability and validity is well established (for a review see Hesse, 2008).

In the present study the German translation of the original English AAI protocol was used (Gloger-Tippelt, 2001). The AAIs in this sample were conducted by the first author and a psychology student after receiving extensive training. Interviews were audio-taped, transcribed and all personal information about the participants was removed from the transcripts. The transcripts were coded by a certified coder^5^. To test reliability 10 randomly selected AAIs were coded by a second certified coder^6^. Coding agreement was 90% (κ = 0.84, *p* ≤ 0.001).

#### Emotional Memory

Subsequently to the AAI assessment participants were asked to memorize and write down three personally meaningful events. In particular, they were successively instructed to describe three single events representing their happiest, saddest and most infuriating personal experience during adolescence (starting by age 10). Descriptions were required to be detailed, including antecedences and outcomes of the situation. Finally, participants were asked to sum up each story in one phrase that was later used as a cue to the emotional experience described (e.g., “death of my grandfather”). These phrases were included in the EEG experiment aiming to help participants to retrieve the emotional memory and, by doing so, trigger the associated affective state.

### Neurophysiological Assessment

#### EEG Procedure

For the EEG assessment, participants were seated in a comfortable chair in a dimly lit, electrically and acoustically shielded cabin. Asymmetrical brain activity was measured during two pseudo-randomized blocks: (1) Resting state EEG asymmetries were measured during two four minute episodes (initial resting state, final resting state), separated by the emotional memory retrieval experiment followed by an ERP paradigm on emotional face processing, which is not included in the current paper. We assessed brain activity during both an initial as well as a final resting state to control whether effects were especially due to the retrieval of emotional memories and thus limited to the intermediating experimental manipulation^7^. During the resting state episodes, participants were instructed to close their eyes, sit quietly and stay calm to avoid movement artifacts. (2) To measure asymmetrical brain activity while retrieving emotional memories, participants were presented with each single phrase cuing their self-reported autobiographical emotional experiences. Phrase stimuli appeared in white fonts on a 19^′′^ black screen with a viewing distance of 115 cm and stimulus presentation was controlled by the experimental software Inquisit (Millisecond Software, Seattle, WA, USA). Each of the three emotional memory retrieval episodes (happiness, sadness, anger) lasted 4 min with phrase stimuli staying on screen the whole time. The order of stimulus presentation was pseudo-randomized. Preceding each of the three emotional memory retrieval episodes a written instruction appeared on screen. More precisely, the instruction page said that participants will be confronted with the phrase they provided during the first assessment and that indicated their happiest, saddest and most infuriating memory, respectively. Moreover, participants were instructed to sit still and to recall the respective situation and, by doing so, they were asked to put their selves back in the corresponding affective state. After reading the instructions, participants could start the respective emotional memory retrieval episode by pressing a button. Throughout the whole assessment participants were monitored via a frontal camera to ensure that they did not move and (during emotional memory retrieval) direct their focus to the screen.

#### EEG Recording

Recording and analyzing of the EEG was performed using BrainVision software (Brain Products, Gilching, Germany). The ERP experiment that was conducted at the same measuring point but not included in the current study required the use of numerous additional electrodes. Thus, a total of 60 active electrodes^8^ based on Ag/AgCl sensors were placed 5 mm in diameter according to the international 10–20 system. To assess eye movements EOG was recorded from electrodes placed below and above the left eye, as well as next to each eye’s outer canthus. The ground electrode was placed at Afz and FCz served as the online reference channel. Signals were acquired using BrainAmp Standard amplifier (Brain Products, Gilching, Germany) that recorded frequencies ranging from 0.016 to 1000 Hz with a resolution of 0.1 μV per bit and a measurement range of ±3.28 mV. They were digitized using a 16 bit A/D converter.

#### EEG Data Reduction and Analysis

For oﬄine processing data were re-referenced to the mastoids and a 115.2 Hz, 24 dB/oct high-cut off filter was applied before downsampling the data to 256 Hz. Saccadic eye movements or eye blinks were corrected according to the Gratton & Cole Procedure (Gratton et al., 1983). EEG-signals were segmented into 2 s intervals. Segments with muscular and other artifacts were removed using a semiautomatic procedure. More precisely, the maximal allowed voltage step was 50.00 μV, the maximal allowed absolute difference of two values in one segment was 300.00 μV and amplitudes were only allowed in the range between –70.00 and +70.00 μV. Also, minimum activity was set to 0.10 μV. In the analysis of artifact-free segments a Hanning window with 50% overlap of each epoch was used to prevent spurious estimates of spectral power. For each resting episode segments were averaged and FFT analyses with a resolution of 0.5 Hz were performed. Subsequently, alpha power (8–13 Hz) was extracted from the spectrum as the sum of according frequency bins. Finally, following a common approach by Coan and Allen (2004) data were log-transformed and alpha power of left electrode sites were subtracted from homologous right sites leading to a frontal (ln[F4]–ln[F3]) and a parietal (ln[P4]–ln[P3]) asymmetry score. These two pairs of electrodes correspond to regions commonly studied in the hemispheric asymmetry literature and, for reasons of comparability, their selection was informed by previous studies (Bruder et al., 1997; Dawson et al., 2001; Coan and Allen, 2003; Shankman et al., 2005; Dennis and Solomon, 2010). It is important to note that since spectral power and neural processing are inversely related, increasing EEG asymmetry scores (referring to relatively increased alpha power in the right hemisphere) are reflective of decreasing RFA and RPA, respectively.

### Statistical Analyses

To compare attachment groups regarding EEG asymmetries during the initial resting state we used repeated measures MANOVAs with attachment (secure, insecure-dismissing, insecure-preoccupied) as the between-subjects factors and region (frontal, parietal) as the repeated factor. Furthermore, for each, the frontal and the parietal region, we conducted analyses to examine the impact of retrieval of emotional memories on EEG asymmetry with regard to attachment representations. Therefore, repeated measures MANOVAs were performed including regional asymmetry scores during both resting state episodes and the three episodes, during which emotional memory retrieval took place. More precisely, condition (initial resting state, sadness, anger, happiness, final resting state) was included as the repeated measure factor and attachment (secure, insecure-dismissing, insecure-preoccupied) was used as the between-subjects factor. All *post hoc* pairwise comparisons were performed using LSD. When the sphericity assumption was violated, degrees of freedom were computed applying Greenhouse–Geisser adjustments to the degrees of freedom.

## Results

### Handedness

We calculated a laterality quotient [(R–L)/(R+L)] as a measure of handedness in accordance with Oldfield (1971). It was confirmed that there were no left-handers in the sample (laterality quotient: *M* = 0.74, *SD* = 0.23), however, one participant’s laterality score equaled zero. Pearson correlations revealed that right-hand dominance was negatively related to frontal EEG asymmetry scores during four out of the five conditions (*rs* ranging from –0.42 to –0.31, *ps* < 0.05). Including the laterality quotient as a covariate did not change the results of our analyses in terms of significance.

### Attachment Representation

Scoring of the AAIs resulted in the following distribution of attachment representations: There were 21 persons with a secure attachment representation and 19 with an insecure one, among the latter 14 persons had an insecure-dismissing and five persons had an insecure-preoccupied attachment representation.

Preliminary analyses showed that attachment representation was not associated with subjects’ age and education. However, there was a significant association between attachment and gender (χ^2^ = 10.3, *p* = 0.006). A closer inspection of the data showed that males were more frequently found in the insecure-dismissing (11 of 14) and less frequently in the secure group (5 of 16), while there was no difference within the insecure-preoccupied group (2 boys, 3 girls). Therefore, gender was used as a covariate in all analyses regarding attachment.

### EEG Asymmetries: Differences between the Attachment Groups

#### EEG Asymmetries in the Initial Rest State

First, we tested whether groups with different attachment representations differed regarding frontal and parietal cerebral asymmetries at rest. The RM- MANOVA with asymmetry scores calculated from brain activity during the initial resting state at each region (frontal, parietal) revealed no main effects neither for region nor attachment. However, there was a significant interaction between attachment and region, *F*(2,36) = 15.65, *p* < 0.001, η^2^ = 0.465.

**FIGURE 1:**
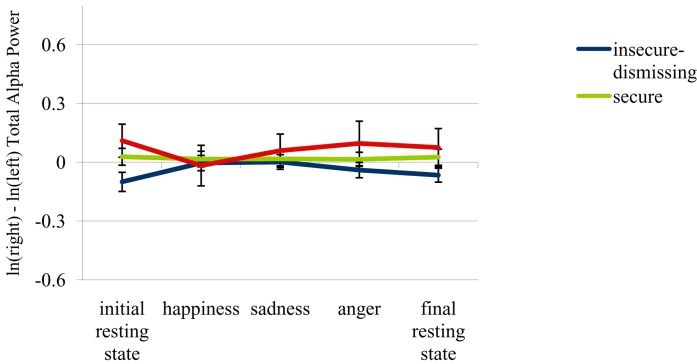
**Frontal EEG asymmetry scores in the insecure-preoccupied, secure and insecure-dismissing attachment group during each condition**. Means and standard errors. Note that episodes inducing happiness, sadness, and anger did not necessarily appear in this order as emotional conditions were counterbalanced between subjects. Lower values are indicative of increased relative right frontal brain activity (RFA).

**Figure 1** shows and LSD *post hoc* comparisons (*p* < 0.05) confirmed, that along with our expectations frontal asymmetry scores for the insecure-dismissing group were decreased and negative (*M* = –0.10, *SD* = 0.18) as compared to the secure group (*M* = 0.03, *SD* = 0.20), *p* = 0.033, as well as to the insecure-preoccupied group (*M* = 0.11, *SD* = 0.19), *p* = 0.026. The mean difference between the latter two groups was not significant. This finding indicates that in comparison to both the other two attachment groups the insecure-dismissing group was more likely to show increased RFA at rest.

**FIGURE 2:**
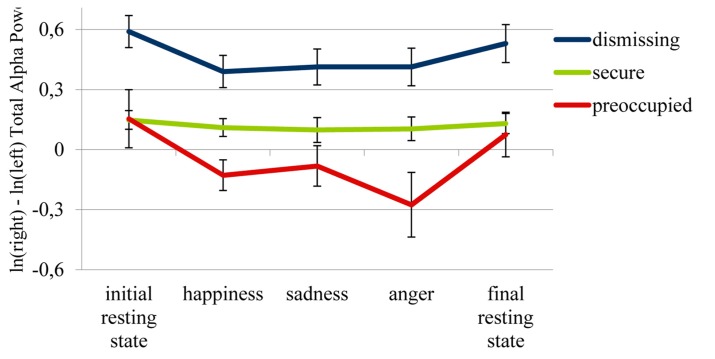
**Parietal EEG asymmetry scores in the insecure-preoccupied, secure and insecure-dismissing attachment group during each condition**. Means and standard errors. Note that episodes inducing happiness, sadness, and anger did not necessarily appear in this order as emotional conditions were counterbalanced between subjects. Lower values are indicative of increased relative right parietal brain activity (RPA).

Regarding the parietal region, LSD *post hoc* comparisons (*p* < 0.05) of the three attachment groups’ EEG asymmetries during the initial resting state revealed the opposite pattern. As can be seen in **Figure 2**, parietal asymmetry scores at rest were significantly increased in the insecure-dismissing group (*M* = 0.59, *SD* = 0.30) as compared to the secure (*M* = 0.15, *SD* = 0.21), *p* < 0.001, as well as to the preoccupied group (*M* = 0.15, *SD* = 0.32), *p* = 0.005, while, again, there was no significant difference between the latter two groups. In other words, at rest, the insecure-dismissing group showed reduced RPA in the parietal region as compared to the secure and the insecure-preoccupied group.

#### EEG Asymmetries during Emotional Memory Retrieval

To test whether emotional memory retrieval differentially impacted frontal asymmetry depending on attachment (see **Figure 2**), a RM-MANOVA with attachment as the group factor was performed on asymmetry scores calculated from frontal brain activity during each of the five conditions. However, there neither were significant main effects nor a significant interaction between attachment and condition.

In the next step, we tested whether attachment groups differed with regard to the impact emotional memory retrieval had on parietal asymmetry scores. Here, the respective RM-MANOVA revealed a main effect for attachment, *F*(2,36) = 8.64, *p* = 0.001, η^2^ = 0.324, which was qualified by an interaction between attachment and emotional condition, *F*(5.09,91.55)^9^ = 3.11, *p* = 0.012, η^2^ = 0.147, indicating that parietal asymmetry scores differentially varied with condition depending on attachment status. These attachment specific patterns are visualized by the graph in **Figure 2**.

**Table 1** shows and LSD *post hoc* tests (*p* < 0.05) confirmed that insecure-dismissing subjects’ parietal asymmetry scores were relatively decreased (inferring an increase in RPA) during each of the emotional retrieval conditions as compared to the initial resting state.

**Table 1:** Parietal asymmetry scores during rest and emotional memory retrieval by attachment group.

Attachment Group	Condition *M (SD)*					*n*
	Initial resting state	Happiness	Sadness	Anger	Final resting state	
Secure	0.15 (0.21)^a^_1_	0.11 (0.20)^a^_1_	0.10 (0.28)^a^_1_	0.10 (0.27)^a^_1_	0.13 (0.23)^a^_1_	21
Insecure-dismissing	0.59 (0.30)^a^_2_	0.39 (0.30)^b^_2_	0.41 (0.33)^b^_2_	0.41 (0.35)^b^_1_	0.53 (0.36)^a^_2_	14
Insecure-preoccupied	0.15 (0.32)^a^_1_	-0.13 (0.17)^bc^_3_	-0.14 (0.22)^bc^_1_	-0.28 (0.36)^b^_2_	0.02 (0.25)^ac^_1_	5

*Means, standard deviations, and statistical significance. Columns: Different Numbers (_1-3_) indicate that the difference between attachment groups was significant within the respective condition, *p* < 0.05 in *post hoc* testing (LSD). Lines: Different letters (^a-c^) indicate that the difference between conditions was significant within the respective attachment group*.

Insecure-dismissing subjects’ parietal asymmetry scores during the emotional retrieval conditions did not differ from each other. However, they were significantly lower during each of the emotional conditions as compared to the final resting state, which -with regard to brain activity- itself did not differ from the initial resting state. Thus, there seems to be a “back-to baseline-recovery” following performance of the emotional task in this group. A very similar pattern emerged for the insecure-preoccupied attachment group, who also showed lower parietal asymmetry scores during each of the emotional conditions (which again did not differ from each other) as compared to the initial resting state. The “back-to-baseline-recovery” in insecure-preoccupied subjects, however, was only significant for the anger condition. In contrast to the two insecure groups, parietal asymmetry scores in securely attached subjects did not indicate any changes in hemispheric activity depending on experimental manipulation. LSD *post hoc* testing confirmed there were no significant differences between any of the five conditions in this group.

In addition to these attachment-related patterns, **Figure 2** also indicates differences between the three groups with regard to hemispheric brain activity across conditions (indicated by the main effect). **Table 1** shows that in the insecure-dismissing group parietal asymmetry scores were significantly higher than in the other two groups during each resting state and each emotional memory retrieval condition (LSD-*post hoc* comparison, *p* < 0.05), except for the difference between the insecure-dismissing and secure group in the anger condition, which did not reach statistical significance. In the insecure-preoccupied group, parietal asymmetry scores were significantly lower than in the dismissing group. These differences were consistent over all conditions. Insecure-preoccupied subjects also showed lower parietal asymmetry scores when compared to the secure group, but only in the happiness and the anger retrieval condition.

## Discussion

In this study, we were looking at attachment-related differences in EEG asymmetries at rest and during the retrieval of emotional memories. Thereby, we focused on both frontal and parietal asymmetries assuming to find differential patterns with regard to attachment in both regions.

### Attachment and Frontal EEG Asymmetry

#### Trait Dependent Frontal EEG Asymmetry

Concerning differences in frontal EEG asymmetries we found a specific pattern of hemispheric activation across the three attachment groups. As expected, we found relatively increased RFA in subjects with an insecure-dismissing attachment representation as compared to those with a secure attachment representation during the initial resting state. In consistency with the approach/withdraw model applied to frontal EEG asymmetry numerous other studies have found relatively increased RFA to be associated with negative affect and withdrawal tendencies (e.g., Hane et al., 2008; also see Davidson, 1992). This interpretation fits well with the phenomenon of insecure-dismissing attachment. According to attachment theory, repeatedly experiencing an irresponsive or rejecting caregiver a child learns to avoid the expression of negative affect (Bowlby, 1969). Assuming stability of the internal working models of attachment from childhood into adulthood, insecure-dismissing adolescents and adults tend to withdraw from negative stimuli as an adaptive strategy to regulate their emotions by, for example, paying less attention to negative stimuli (Hazan and Shaver, 1994; Simpson et al., 1996; Cooper et al., 1998). In line with our finding, Rognoni et al. (2008) found an increase in resting RFA in avoidantly attached subjects as assessed via self-report. Also, Dawson et al. (2001) found insecurely attached children to show decreased left frontal asymmetry as compared to securely attached ones. Taken together our findings provide further evidence for a stable pattern of greater withdrawal (or less approach) motivated tendencies in insecure-dismissing subjects to show on a neurophysiological level. Noteably, there is strong evidence that this frontal hemispheric pattern is associated with emotional disorders like depression in infants and adults (for a review see Thibodeau et al., 2006).

Regarding the insecure-preoccupied group we did not find an elevated RFA, which is in contrast to Rognoni et al.’s (2008) study, who found this pattern in both insecure as compared to the secure groups. However, we found insecure-preoccupied subjects to show relatively increased LFA during rest. Even though the difference to the secure group failed to reach significance (perhaps partially due to the small size of the sub-sample) this finding points in the right direction as it is theoretically well founded. Insecure-preoccupied subjects tend to hyper-activate the attachment system and report about childhood experiences eliciting highly intense emotions. Indeed, left frontal hemisphere activation is associated with approach-related motivation, coping and proactive social behavior (Davidson, 1992; Master et al., 2009; Licata et al., 2015). However, relatively increased LFA has also been found in dispositional anger which can be regarded an approach related tendency as well (Harmon-Jones and Allen, 1998). Thus, insecure-preoccupied subjects’ frontal asymmetry scores at rest may indicate that they are more prone to approach rather than withdraw from emotional challenges. However, this tendency may be rather accompanied by negative emotions like anger, which may contribute to the assumption that attempts to resolve their entanglement rather tend to fail.

The secure group’s frontal asymmetry scores were distributed around zero indicating rather symmetrical brain activity during rest. This is in agreement with Rognoni et al.’s (2008) findings. It could be interpreted in light of securely attached persons’ flexibility to differentially react to various environmental challenges without being predisposed to withdraw or approach.

#### State Dependent Frontal EEG Asymmetry

While attachment-related differences in frontal asymmetry could be well identified during rest, significant differences between attachment groups could not be found during phases of emotional memory retrieval intended to induce specific affective states. It could be assumed that attachment differences in frontal asymmetry scores rather represent a trait variable and thus are less affected by the experimentally induced affective state. Indeed, frontal asymmetry have shown to be unaffected by emotional valence in other studies using emotional tasks as well (e.g., Hagemann et al., 2005; Feng et al., 2012; Mennella et al., 2015). Moreover, in the current study the main effect for attachment diminished for frontal asymmetry during the retrieval of emotional memories. Thus, it may also be that the high emotional involvement induced by all three types of memories activated and deactivated the approach/withdrawal system in a more complex way obliterating previous differences between attachment groups. Future research may address this issue by, for example, examining whether the nature of the emotional memory may account for this non-finding. It could be possible, that specifically focusing on attachment relevant memories is a more powerful approach to elicit differences in emotional memory processing with regard to attachment security.

### Attachment and Parietal EEG Asymmetry

#### Trait Dependent Parietal EEG Asymmetry

Regarding the parietal region, we found that, during a resting state, insecure-dismissing subjects elicited significantly higher asymmetry scores as compared to secure and insecure-preoccupied subject (reflecting decreased RPA in insecure-dismissing individuals) which is in line with our hypothesis. Such right-sided parietal hypo-activity has repeatedly been related to reduced arousal and low emotionality (Shankman et al., 2005; Hayden et al., 2008). This neurophysiological evidence fits with the assumption of insecure-dismissing subjects to be predisposed to show inattentiveness to emotional stimuli. Concluding from our findings this inattentiveness may result from a larger threshold to be emotionally affected due to trait hypo-arousal, which is in line with Kobak and Sceery (1988), who claimed that the insecure-dismissing attachment pattern is characterized by hypo-activation of emotions.

Notably, it has been suggested that relatively decreased RPA represents an endo-phenotype for depression (Bruder et al., 1997; Kentgen et al., 2000; also see Stewart et al., 2011). Indeed, as shown in an overview by Dozier et al. (2008) there is abundant evidence linking insecure attachment to the development of affective disorders in adulthood. This also includes the insecure-dismissing state of mind. Moreover, Duggal et al. (2001) reported that insecure attachment (both resistant and avoidant) predicted depression in adolescence. According to Fonagy et al. (1996) parents of depressed persons were rated as less supportive and more rejecting. Taking the developmental perspective, experiences with an irresponsive and rejecting caregiver may lead to an inner working model of the self as not loved and unworthy and may hinder the child to develop appropriate emotion regulation strategies. Low self-esteem as well as a restricted ability for emotional regulation are regarded as risk factors for the development of depression (e.g., McCauley et al., 2001). Thus, our finding on decreased RPA (as well as increased RFA) in insecure-dismissing subjects may represent the biological substrate that signifies vulnerability to depression in these individuals.

While decreased RPA during rest was found in the insecure-dismissing group, individuals with a secure and insecure-preoccupied attachment representation did not differ with respect to parietal asymmetry during rest. This similarity between secure and insecure-preoccupied attachment groups applies to our findings on frontal asymmetry as well, indicating a common trait-like emotional system in both groups that clearly differs from insecure-dismissing subjects. According to Kobak and Sceery (1988) a main difference between insecure-dismissing and preoccupied persons relates to the activation of emotions. While the insecure-dismissing pattern is characterized by emotional hypo-activation, hyper-activation is typical for the insecure-preoccupied pattern. Thus, although pre-occupied persons have difficulties to regulate emotions appropriately, a high sensitivity or attentiveness to emotional information may be assumed for them comparable to secure persons.

Regarding this assumption our findings can be interpreted in line with the “right-hemisphere hypotheses” that claims that the right hemisphere is especially involved in the automatic generation of emotional responses (Gainotti, 2000; Hagemann et al., 2005; for a review see Borod, 1993). Testing the hypotheses, Hagemann et al. (2005) found greater right-sided activity to be associated with the intensity of felt emotions and that this cortical activity pattern was especially evident in the parietal region.

Thus, it might be that our finding on increased resting RPA in secure and insecure-preoccupied as compared to insecure-dismissing individuals indicates a trait-like sensitivity to emotional information while their decreased RFA reflects both their tendencies to explore affective states instead of using withdrawal strategies.

#### State Dependent Parietal EEG Asymmetry

As expected, we found emotional memory retrieval to affect parietal hemispheric asymmetry, but again, this finding was given irrespective of emotional valence. Thus, the valence or type of the retrieved memory did not systematically affect brain processing in the parietal region and inter-individual differences of attachment groups remained relatively stable or –with regard to the insecure-preoccupied group- became even more pronounced (at least for anger and happiness). Regarding the “right-hemisphere hypotheses” mentioned above, our non-findings of an effect of emotional valence are in line with empirical evidence that suggests that impairments in the right cortical region have tremendous effects on both positive and negative emotional responses (for a review see Borod, 1993).

More interestingly, there appeared to be a shift towards lower parietal asymmetry scores during the emotional memory retrieval task in both insecure groups but not in the secure group. Interestingly, a comparable shift has also been found in anxious individuals during a fear inducing narrative task (Heller et al., 1997). This decrease in right-sided alpha power is reflective of an increase in RPA that can be interpreted as enhanced arousal (Heller et al., 1997; Metzger et al., 2004). Notably, we found that by the final episode at rest (following the retrieval of emotional memories), parietal asymmetry scores in insecure subjects have increased again showing this specific pattern of brain activity to be clearly state-dependent.

Our findings suggest that parietal asymmetry during emotional memory retrieval may be indicative of one’s ability to effectively regulate emotions. Both insecure attachment groups characterized by a restricted ability to regulate emotions, albeit in a very different way, show an increase of RPA, that could not be observed in the secure subjects characterized by high emotional regulation capacities. Interestingly, during the retrieval of happiness and anger (but not sadness) evoking memories, the difference between the insecure-preoccupied and the secure group reached significance. As we assume both of these affective states to be more likely to elicit increased arousal as compared to sadness, they seem to be more powerful in the detection of trait-dependent arousal-related cerebral differences between attachment groups.

In sum, these findings lead to the conclusion that in our study insecurely attached individuals were more affected by the memory retrieval task, probably because confrontation with highly salient emotions provided a greater challenge to their restricted emotion regulation capacities. Interestingly, both insecurely attached groups, the insecure-dismissing as well as the insecure-preoccupied one, showed this shift, even though they started at different baselines. Thus, they may differ from one another with respect to attention and sensitivity to emotional information, however, when directly confronted with personally highly salient emotions induced by experimental induction, on a neurophysiological level, they appear to respond very similar, as both their regulation strategies are rather ineffective. Interestingly, Beijersbergen et al. (2008) found that while during the AAI insecure-dismissing attached adolescents were capable of remaining a physiological state that was comparable to that of secure ones, their emotion regulation capacities were highly challenged during a family interaction task as indicated by increased heart rate reactivity. Thus, including our findings it can be suggested that the effectiveness of emotion regulation strategies typical for insecurely attached individuals are strongly depends on the nature of the emotional challenge.

The pattern of the securely attached group’s hemispheric brain activity during emotional memory retrieval is quite different. According to attachment theory they are expected to be sensitive and attentive to emotional information, still, emotional memory retrieval did not affect parietal hemispheric activity (no increased arousal) in this group. Indeed, this may be due to their high capacity to regulate emotional states. Equally important, securely attached individuals by theory are capable of freely evaluating meaningful experiences and report them in a coherent manner. In a recent study, Spangler and Zimmermann (2014) found young adolescents with a history of secure attachment to be more aware of their emotions as compared to subjects with an insecure attachment history. Furthermore, they were found to be more capable of communicating affective states as well as using social emotion regulation strategies. These competencies may lead to a more effective integration of highly salient experiences, and thus, may account for their well-regulated affective state during emotional memory retrieval. Finally, emotional memory retrieval in our study may have been less challenging for securely attached individuals.

Taken together, it seems that, while the differences in parietal asymmetry during resting state reflect differences between the attachment groups with respect to sensitivity to emotional information, the differences in parietal asymmetry during emotional memory retrieval appear to rather reflect differences in emotion regulation capabilities specific for the attachment groups.

Regarding our methodological approach of including both the frontal and parietal asymmetries in our analyses, it is noteworthy to mention, that we have found effects of attachment to be more pronounced at parietal sites and that the right hemisphere seems to play a prominent role in affect regulation, which has been suggested and discussed in other studies (see Hagemann et al., 2005).

## Conclusion

Summing up, differences in attachment representations were found to affect both the frontal and parietal organization of hemispheric asymmetry at rest and (for parietal region only) during the retrieval of emotional memories, however, irrespective of valence.

More precisely, during a resting state we found insecure-dismissing subjects to show increased RFA and at the same time a decrease in RPA. This finding on trait-like hemispheric asymmetries in insecure-dismissing subjects corresponds to their disposition to show –on the one hand- tendencies to withdraw rather than approach, and –on the other hand- lower state-like arousal in comparison to the other attachment groups. Notably, this specific pattern in hemispheric asymmetries has also been found to be stable in dysphoric individuals (Mennella et al., 2015), and thus, may indicate of an increased vulnerability to depression in insecure-dismissing subjects.

Moreover, when compared to the secure group, insecure-dismissing and insecure-preoccupied subjects showed reduced and enhanced state-dependent arousal, respectively, as indicated by parietal asymmetry scores during emotional memory retrieval. At the same time, both insecurely attached groups seemed to rather use state-dependent dysfunctional strategies to regulate affective states related to personally highly salient emotional experiences. This assumption was concluded since when unlike securely attached individuals they showed a clear shift in RPA towards increased arousal during the emotional task. These specific neurophysiological substrates indicating less effective emotion regulation may again be viewed as a vulnerability to develop an affective disorder in insecurely attached individuals.

## Limitations

The strength of this study lies in the application of an emotional imagery task that we assume a valid measure to probe the neural substrates of emotion regulation as subjects chose upon a personally highly salient emotional memory. However, it is crucial to note that there was no control condition, in which participants were exposed to an autobiographical episode with a neutral valence. Still, it may be hard to isolate a salient neutral memory that is not contaminated by any emotional tone. This is why we chose to compare neural activation during emotional memory retrieval with pre- and post-measurement resting state conditions. In future studies, it could be useful to differentiate between attachment related and non-attachment related emotional memories. Thereby, one may be more successful in eliciting attachment related differences in frontal hemispheric asymmetry during the processing of emotional information. Such approach would also shed light on the generalizability of our findings to attachment related differences in emotion regulation in late adolescence. Furthermore, even though the distribution of attachment classification is in agreement with meta-analytic findings (Van IJzendoorn, 1995), our analyses are restricted to reduced statistical power with regard to the small sample size, in particular that of the insecure-preoccupied group. Also, since this was the first study of its kind, future studies are needed to replicate and potentially extend our findings. Another limitation of this study is that the design does not allow to draw upon assumptions on the causal mechanisms between neural measures and emotion regulation strategies associated with different attachment representations. While associations between frontal asymmetry and attachment as well as maternal depressive symptoms have already been found in young children (e.g., Field et al., 1995; Dawson et al., 1997; Dawson et al., 2001), a recent study’s findings suggest that frontal hemispheric asymmetry may be less influenced by environmental factors (Licata et al., 2015). It would be very interesting to apply a longitudinal approach to attachment related emotion regulation strategies and the neural circuits that are associated with the phenomenon beyond childhood.

## Ethics Statement

The study was conducted in accordance with the Code of Ethics of the German Psychological Association (from 09/28/2004), which is essentially based on the Code of Ethics of the APA (Ethical Principles of Psychologists and Code of Conduct, American Psychologist, 2002, 57, 1060–1073). According to the rules of the German Research Foundation, it was not required to apply for a formal vote for this study, because (1) the participants were healthy (no patient groups), (2) no invasive methods were used, and (3) their participation did not present any risk to the participants. Finally (4), for studies using ERP assessments, a formal vote is only required if subjects’ age is either below 14 or above 65 years.

The participants were informed about the study’s aims and methods. In addition, they were informed that (1) their participation is voluntary and that they can withdraw from it any time without stating reasons, and (2) that the data were treated according to the data protection law and saved anonymously. Each participant signed the informed consent form before participation.

## Author Contributions

MK: collection, design, analysis and interpretation of data, literature research, writing. RL: concept, design, collection, analysis, and interpretation of data, literature research. GS: concept, design, supervision, writing, critical review.

## Conflict of Interest Statement

The authors declare that the research was conducted in the absence of any commercial or financial relationships that could be construed as a potential conflict of interest.

## Footnotes

1 Note that the terms “-ambivalent” and “-avoidant” apply to studies using self-report measures but refer to the terms “-preoccupied” and “-dismissing”, respectively, that are commonly used in studies using narrative measures of attachment.

2 Alpha power is an indicator of neural deactivation (Lindsley and Wicke, 1974), and thus, speaking about brain activity in a given hemisphere, the alpha power score needs to be inverted.

3 See Leyh et al. (2016b) for a description of the third laboratory assessment.

4 The description of the AAI-procedure used here was drawn from Leyh et al. (2016b).

5 Johanna Behringer, Institute of Psychology, University of Erlangen-Nuremberg, Germany.

6 Kathrin Beck, Institute fuer Therapieforschung, Munich, Germany.

7 We are aware that the collection of ERP data on emotional face processing may have had effects on brain activity during the final resting state. However, our results show that brain activity in the final resting state did not differ from the initial resting state with regard to statistical significance (see Results), suggesting there were no spill-over effects due to the second paradigm.

8 AF4, AF8, C1, C2, C3, C4, C5, C6, CP1, CP2, CP3, CP4, CP5, CP6, CPz, Cz, F1, F2, F3, F4, F5, F6, F7, F8, FC1, FC2, FC3, FC4, FC5, FC6, FT10, FT7, FT8, FT9, Fz, O1, O2, Oz, P1, P2, P3, P4, P5, P6, P7, P8, PO10, PO3, PO4, PO7, PO8, PO9, POz, Pz, T7, T8, TP10, TP7, TP8, TP9.

9 Degrees of freedom corrected according to Greenhouse–Geisser.
